# Design of a Plantar Pressure Insole Measuring System Based on Modular Photoelectric Pressure Sensor Unit

**DOI:** 10.3390/s21113780

**Published:** 2021-05-29

**Authors:** Bin Ren, Jianwei Liu

**Affiliations:** Shanghai Key Laboratory of Intelligent Manufacturing and Robotics, School of Mechatronic Engineering and Automation, Shanghai University, Shanghai 200444, China; jianweiliu@shu.edu.cn

**Keywords:** optical sensing principle, modular sensing unit, plantar pressure measurement, gait parameters

## Abstract

Accurately perceiving and predicting the parameters related to human walking is very important for man–machine coupled cooperative control systems such as exoskeletons and power prostheses. Plantar pressure data is rich in human gait and posture information and is an essential source of reference information as the input of the exoskeleton control system. Therefore, the proper design of the pressure sensing insole and validation is a big challenge considering the requirements such as convenience, reliability, no interference and so on. In this research, we developed a low-cost modular sensing unit based on the principle of photoelectric sensing and designed a plantar pressure sensing insole to achieve the purpose of sensing human walking gait and posture information. On the one hand, the sensor unit is made of economy-friendly commercial flexible circuits and elastic silicone, and the mechanical and electrical characteristics of the modular sensor unit are evaluated by a self-developed pressure-related calibration system. The calibration results show that the modular sensor based on the photoelectric sensing principle has fast response and negligible hysteresis. On the other hand, we analyzed the area where the plantar pressure is densely distributed. One benefit of the modular sensing unit design is that it is rather convenient to fabricate different insole solutions, so we fabricated and compared several pressure-sensitive insole solutions in this preliminary study. During the dynamic locomotion experiments of wearing the pressure-sensing insole, the time series signal of each sensor unit was collected and analyzed. The results show that the pressure sensing insole based on the photoelectric effect can sense the distribution of the plantar pressure by capturing the deformation of the insole caused by the foot contact during locomotion, and provide reliable gait information for wearable applications.

## 1. Introduction

In recent years, much research on lower limb exoskeleton robots has been carried out [1,2,3,4] to help with human activities and enhance the functions of the human body. Among many exoskeleton/prosthetic assist devices, the primary task is to provide the wearer with assistance in walking motions. The detection and sensing of data information related to human motion is the basis of and key to the compliance control of the lower limb wearable device [5,6]. The human wearer is the controlling center of an exoskeleton system. The real-time information on the human body is the primary source of the exoskeleton man-machine coupling control system, which accurately senses and predicts the state of human walking. An exoskeleton controller can detect the intent of the motion and control the corresponding parts of the drive module by sensors. At the same time, the comparison between the human body and the exoskeleton motion is analyzed to provide feedback to ensure that the exoskeleton can respond to human action quickly and accurately. It is also vital to provide a safety guarantee for the human body in the human–machine coupling system.

The interactive contact between the feet and the ground is the most intuitive manifestation of human motion dynamic information. Plantar pressure data contains abundant human gait and posture information [7]. In the initial stage, the fixed system [8,9] (such as motion capture system, force measurement platform system, etc.) is used to provide a simple and effective way to explore the basic biomechanical laws walking process. However, these non-mobile systems can only be used in limited space and usually have expensive construction and maintenance costs [7]. In practical applications, when people need to wear mobile assistive devices to cope with various environments or terrains in an outdoor environment, the motion perception system requires efficiency and portability. Thus, pressure-sensitive insoles/socks provide a better trade-off. They usually use flexible materials as their medium (such as silicone [10], fabric [11], composite materials [12], etc.), employing different sensing principles (for example, piezoresistive, capacitive, piezoelectric, etc.) for the portable wearable plantar pressure measurement system to collect information on the movement of the portable robot. Powerful technical support is provided in the wearable application.

There are some commercialized sensing insoles based on different sensing principles. The F-Scan system (Tekscan^®^, South Boston, MA, USA) [13] uses FSR (Force-sensing resistors) sensors, the ParoTech system (Paromed^®^, Neubauer, Germany) [14] uses piezoresistive sensors and the Pedar system (Novel^®^ GmbH, Munich, Germany) [15] uses an embedded capacitive sensor. In addition to the commercial insole design, researchers are still trying to innovate in structural layout and processing algorithms. Liu et al. [16] designed a pressure-sensitive foot for the lower extremity exoskeleton. The pressure-sensitive foot can measure plantar pressure to sense the contact with the ground and reflect the wearer’s behavioral intentions. Lim et al. [17] compared the three flexible pressure sensors of FSR, FlexiForce and capacitive sensors. They chose the FlexiForce sensor to design the pressure insole and detect the gait phase based on the threshold segmentation method of the pressure center. Wu et al. [18] used an insole made of three FSR sensors to detect four gait sub-phases. Chen et al. [19] used FlexiForce sensors to design a pair of insoles with eight sensors to identify walking patterns. Zhang et al. [11] developed a simple, low-cost and highly integrated insole based only on fabric for measuring plantar pressure, the principle of which mainly relies on the capacitive mechanism. However, as emphasized in the paper [20], it is precisely because of the light, thin and soft characteristics of these sensing units that they will produce unpredictable distortion and deformation on the contact surface, making the sensing response unable to be accurately estimated. What is more, this type of sensor usually needs to go through an additional modulation circuit to amplify the signal.

In addition to those plantar pressure-sensing insole solutions based on membrane-based sensor units mentioned above, the research teams tried to develop their pressure-sensing insoles to provide more reliable plantar pressure information sensing solutions. Park et al. [21] showed a novel use of high-sensitivity crack-based strain sensors to make plantar pressure insoles. The technical solution based on photoelectric induction has attracted the attention of many researchers. Leal et al. [12,22] grasped the characteristics of polymer optical fiber (POF), such as lightweight, anti-magnetic and electrical isolation, and initially designed and integrated four POF of the sensing unit [12] to monitor the ground reaction force during the gait, and the follow-up research work [22], combined with the advantages of 3D printing technology rapid prototyping, developed customizable pressure sensing insoles and increased the number of POF sensors to 15. The research team from Santa Ana [20,23,24] used the light-emitting unit and the photosensitive unit arranged on the same side and realized the sensing of pressure to electric signal with the help of an elastic rubber cover covering the sensor element, and applied this technology to the outside, in the interactive signal perception between the bones and the human body in the ring. These technical solutions paved the way for the research on a more stable and comprehensive plantar pressure sensing system.

In this paper, we mainly completed the following work: firstly, a modular pressure sensor based on photoelectric sensing technology is properly designed and fabricated. The components of the sensor are from commercially available materials. Its structural design is novel, and no additional signal amplifier is needed in the sensing acquisition circuit to capture the sensing signal. In the manuscript, we introduced how to use easily accessible, low-cost manufacturing methods and materials to make such a modular sensor so that other researchers can easily reproduce technology and further carry out related research work. Secondly, the designed modular pressure sensor is implemented on a specially designed programmable control calibration instrument [25,26]. The mechanical and electrical characteristic evaluation experiment proved that the modular sensor has specific applicability in pressure sensing. Thirdly, based on the analysis of the pressure distribution area, two different sensor layout schemes were specified, and the modular pressure sensor was integrated into the pressure sensing insole. The performance of two insole solutions were compared in the preliminary experiment. Finally, combined with the dynamic walking experiment, the performance of the manufactured pressure sensing insole in the application of collecting plantar pressure was explored, and the results showed that the insole system could monitor in real-time plantar pressure and provide reliable gait-related parameters, which provides potential value in wearable walking robot equipment, exoskeleton, power prosthetics and other applications.

## 2. Materials and Methods

### 2.1. Modular Pressure Sensing Unit

#### 2.1.1. Sensing Principle

The sensor technology used in this research mainly relies on the photoelectric effect. That is, the photoresistor exhibits different resistance characteristics under different ambient light intensities. The resistance of the photoresistor decreases as the incident light (visible light) increases. Under normal conditions, its resistance can reach 10,000 to 10 million ohms while, under photosensitive conditions (such as 100 Lux), its resistance is only a few hundred to a few thousand ohms.

**Figure 1:**
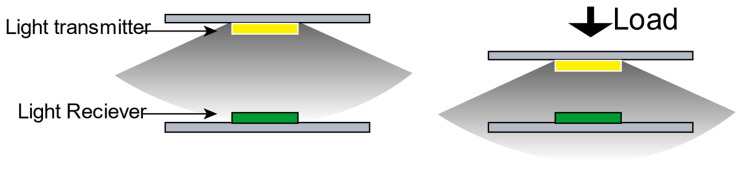
Photoelectric sensing principle diagram.

Generally, when the walking foot touches the ground, the sole exerts a force on the ground through the insole and the shoehorn. During this period, the insole undergoes a certain degree of deformation in the direction perpendicular to the contact surface. We hope to use this tiny deformation relationship to induce the induction between the photodiode and the photoresistor. In other words, during the deformation process, the distance between the light emitter (light-emitting diode) and the light receiver (photoresistor) changes to cause a change in light, which in turn triggers a change in the resistance of the photoresistor, as shown in Figure 1. We use photoelectric technology to capture the slight deformation of the insole caused by plantar pressure. It is crucial to design an appropriate design structure to integrate the light-emitting diode and photoresistor into a narrow space with the limited thickness of the insole and provide a light-transmitting medium with suitable material properties.

#### 2.1.2. Design and Manufacturing

Compared with the overall layout, the modular sensing solutions are convenient for customizing. Therefore, we designed a modular sensing unit based on the sensing principle introduced above, adjusting the sensor layout according to different foot sizes to embed pressure sensing insoles. The designed modular sensing unit is mainly composed of three parts: (1) a flexible circuit board containing a photodiode and a photoresistor; (2) an elastic light-transmitting silica gel medium used to absorb the applied pressure and recover when the pressure is removed; (3) some necessary electrical connections.

(a) Flexible circuit board

**Figure 2:**
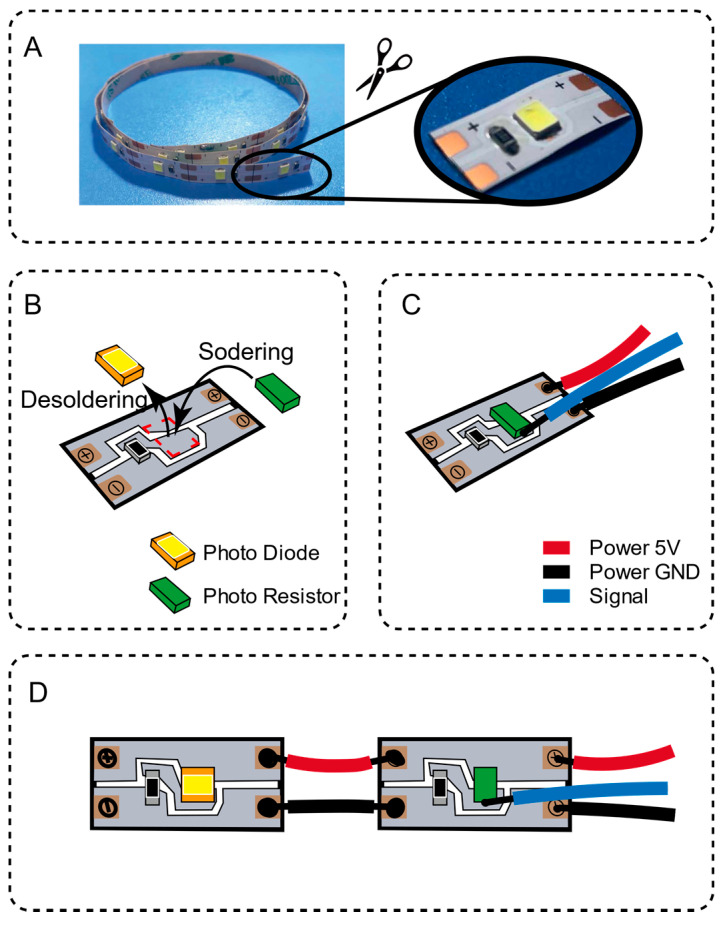
Fabrication of flexible circuit board inside the sensing unit. (**A**) cut out a single LED unit from the light strip; (**B**) replace the LED lamp beads with a photoresistor; (**C**) weld the power supply and signal wires; (**D**) weld the LED strip and the photoresistor strip together.

Optical transmitters and optical receivers play an essential role in optical sensing technology. In this article, we introduce a low-cost method to use this technology. The light-emitting element and the photosensitive element are obtained by modifying the commercial LED strip (Telesky, Shenzhen, China), as shown in the figure. The LED strip is based on a flexible printed circuit board (FPC), a photodiode powered by 5 V and a corresponding current limiting resistor. Each light-emitting diode is independently powered and can normally work when the anode and cathode are connected to a 5 V power supply. It is worth noting that the applicable model of the photodiode is 5050 (5 mm × 5 mm), which means that the LED footprint can fully accommodate the 1206 SMD surface mount package. Therefore, according to the positive and negative polarity, we chose a 1206 SMD surface mount package type photoresistor, replacing the lamp beads in the LED light strip. At the same time, we soldered a signal wire from the voltage divider circuit node and led it out. After using a jumper wire to connect the LED light bar and the photoresistor bar, we used hot melt glue to cover the soldering point to improve its reliability, as shown in Figure 2.

(b) Flexible light-transmitting medium

The pressure sensing unit needs to tolerate a specific pressure range and return to the original state when the applied pressure is released. As an inexpensive material, organic silicon materials are widely used in the design of many flexible sensors. This article uses semi-transparent silica gel (Beijing Hibas Technology Co., Ltd., Beijing, China) as the primary elastic material and is mixed with corresponding plasticizers to catalyze the solidification process of silica gel. In the initial prototype design, we found that only silica gel was used to prepare a light guide medium with higher hardness, which made the signal not obvious enough for us in the plantar pressure range of interest. To optimize the design of the sensor unit, we have included a softener (dimethazone). By mixing different proportions of silica gel and softener, we finally determined the appropriate ratio of the mixture as the elastomer medium, and its weight ratio is silica gel: softener = 4:1.

(c) Integration process

**Figure 3:**
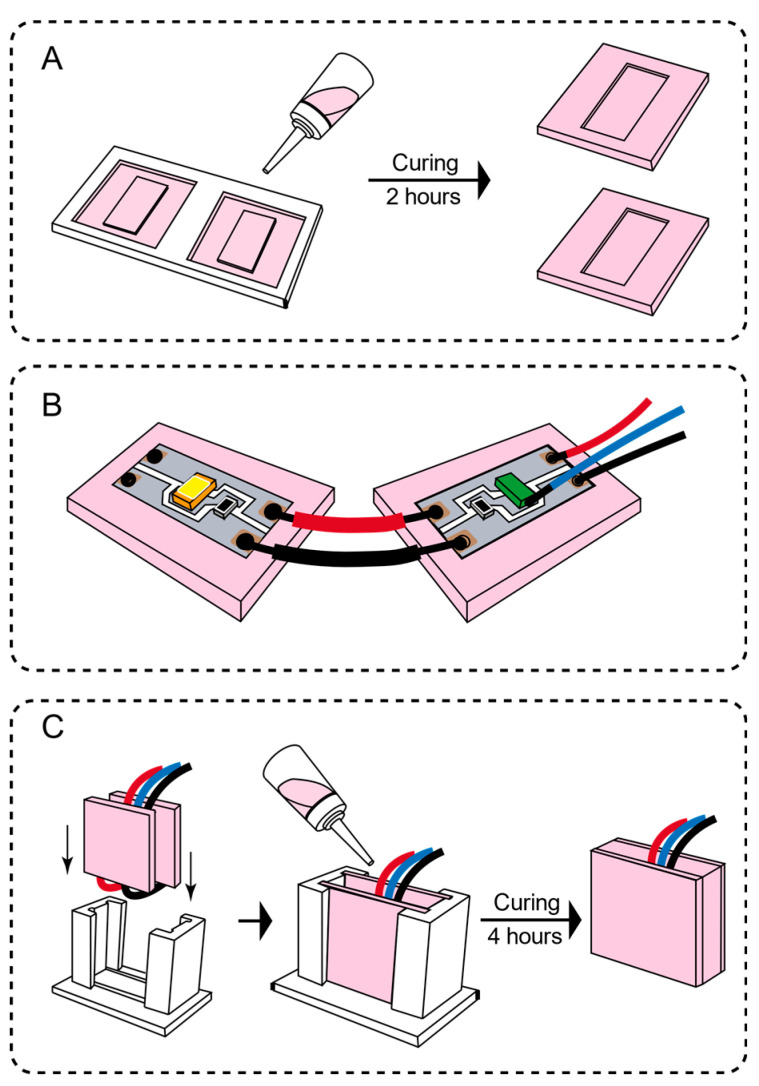
The integration process of the flexible module of the sensing unit. (**A**) Silica gel baffle pouring (**B**) The sensor unit is fixed on the silica gel baffle (**C**) Put into the mold and add the silica gel mixture to wait for solidification.

**Figure 4:**
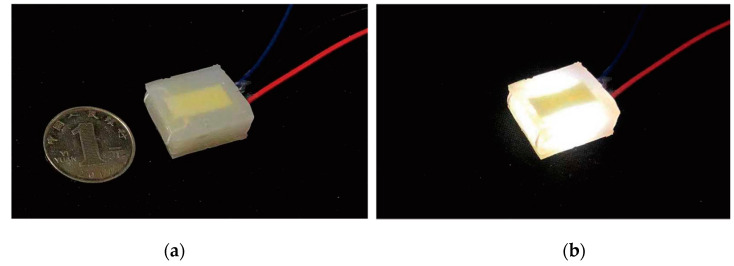
The manufactured sensing unit. (**a**) unit without power (**b**) unit with power.

After preparing the circuit and the elastic medium, we used Autodesk Fusion 360 modelling software to design multiple molds for casting and used FDM3D printers to prepare the molds. As shown in Figure 3, one of the molds is used to make the sensor unit’s silicone shell baffle (thickness 1 mm). The other mold is used to integrate the entire sensor unit (including the silicone shell baffle, circuit and elastic medium). Figure 3B shows the process of fixing the sensing unit circuit in the grooves of the two silicone shell baffles and, finally, integrating it into a whole with the cured silicone medium. The final size of the sensor is a square flexible sensing unit which is 20 mm in width, 20 mm in length and 7 mm in height. One single sensing unit weighs 2~2.2 × 10^−3^ kg (silica gel density 700 kg/m^3^). Figure 4 shows the manufactured sensor with and without power supply status.

#### 2.1.3. Characteristic Analysis System

**Figure 5:**
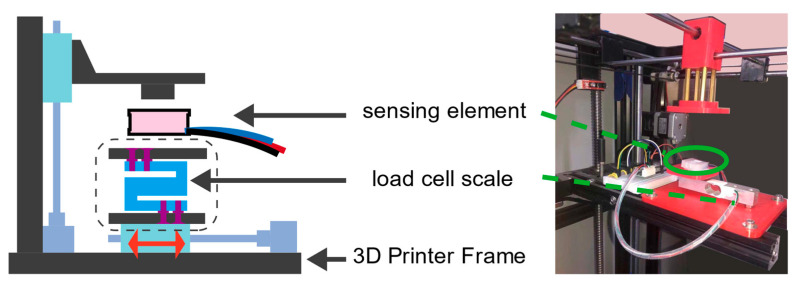
Characteristic analysis system.

We analyzed the mechanical and electrical characteristics of the manufactured modular sensing unit and calibrated the mapping relationship between the quasi-static pressure and the output signal. It is necessary to conduct a characteristic evaluation experiment on the sensing unit before being integrated into the insole. In the paper [19], the author provides a low-cost calibration method that allows researchers to carry out the calibration test of pressure-related sensing units without using expensive calibration equipment. Here, we introduce an improved version. The calibration analysis system is shown in Figure 5. First, we established a calibration instrument according to the process described in [19] to measure the load force and deformation during the static load test. The calibration instrument is a microsystem composed of three parts: (1) HX711 force measurement unit, on both sides of which are bolted 3D printed rigid plastic (PLA) boards; (2) HX711 amplifier circuit module; (3) an Arduino NANO microcontroller for collecting and recording data from the measuring instrument; secondly, we replaced the original printing platform of the FDM3D printer with a load cell. At the same time, the print head of the printer was replaced with a corresponding contact pressure head according to different test purposes. The loading and unloading can be designed by writing G-code control codes for the 3D printer test. In other words, compared to manually adding weight, the mechanical frame of the 3D printer makes the process more controllable.

### 2.2. Insole Solution

#### 2.2.1. The Layout of Insole

**Figure 6:**
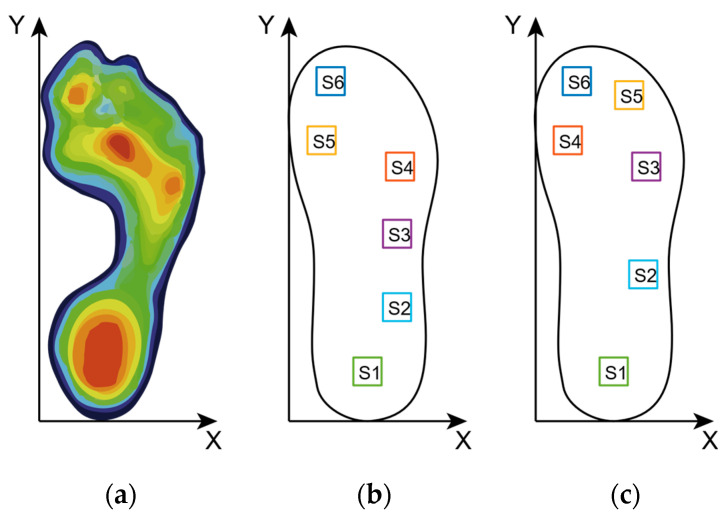
The plantar pressure distribution. (**a**) typical plantar pressure distribution in a standing position; (**b**) first insole layout solution; (**c**) second insole layout solution.

This part mainly introduces the design and production of flexible pressure sensing insoles based on the photoelectric pressure sensing unit designed above. The most challenging problem is that the layout of the sensing unit in the pressure sensing insole needs to fully consider the plantar pressure distribution. From the intuitive impression, due to the irregular surface of the sole and the dynamically changing contact position, the pressure is not evenly distributed on all the surfaces of the insole. For example, the pressure on the inside of the foot arch is slight, while the heel and forefoot areas have greater pressure. Figure 6 is a diagram of plantar pressure distribution in the standing state from reference [13]. From the heat diagram, it can be observed that the plantar pressure is mainly distributed in the heel, forefoot and toes, among which the force in the toe area is mainly located on the thumb. Therefore, placing sensors in these locations can provide more relevant data on plantar pressure.

Because the difference in the foot size varies among different people, and the layout of the pressure-sensing insole does not have a proper guideline, two preliminary steps were made based on the author’s foot size. A flexible pressure insole solution, as shown in the figure: the layout of the sensor mainly refers to the aforementioned plantar pressure distribution. In the first solution, sensors are placed in six places: the first toe, the third toe, the first metatarsal, the fifth metatarsal, the outside of the arch of the foot and the heel. In the second solution, sensors are placed in six places: the first toe, the first metatarsal, the fifth metatarsal, two outside of the foot’s arch and the heel. “S1” in Figure 6b, c represents sensor 1, “S2” represents sensor 2, and so on.

#### 2.2.2. Insole Manufacturing

**Figure 7:**
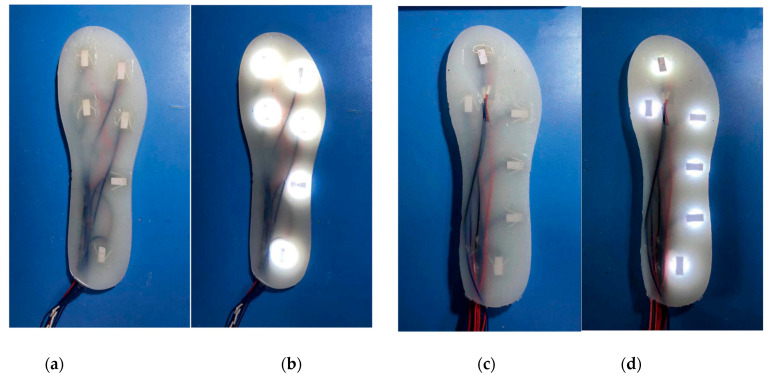
Two insole solutions for the right foot insole. (**a**) Insole solution No. 1 without power (**b**) Insole solution No. 1 with power (**c**) Insole solution No. 2 without power (**d**) Insole solution No. 2 with power.

The manufacturing process of the insole is as follows: first, we designed and 3D printed the casting mold for the insole (right foot), which is size 43 according to Chinese standards; secondly, the sensor unit was fixed in the insole casting mold according to the corresponding position of the two insole layout solutions (six sensors for each solution); all the wires are guided to the outlet at the heel of the casting mold and fixed in the free position where the sensor does not interfere with each other; after the mold outlet is closed with a 3D printed lid, the silica gel mixed with the same proportion softener (dimethazone) was poured into the casting mold—it should be noted that the cavity height of the insole casing mold is 0.5 mm higher than the height of the single sensing unit which ensures the surface of the insole after casting is as flat as possible; finally, the mixed silica gel takes 4 h to solidify, and all the power supply wires are welded into a bus for external power supply. The weight of the two insole solutions are 136 g and 132 g, respectively. Figure 7 shows the two plantar pressure-sensing insole solutions in the non-powered state and the powered state.

#### 2.2.3. Electrical System

**Figure 8:**
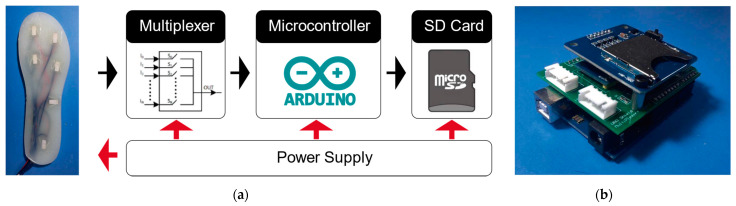
Circuit system diagram (**a**) and Arduino UNO expansion integrated circuit board (**b**).

To measure the sensor’s signal and record the data, we designed the circuit system according to the system framework shown in Figure 8. The circuit system is mainly used for sensor signal acquisition, data preprocessing and data storage, including a microcontroller module, a data storage module and a power supply device. Since the voltage divider circuit of the sensing unit has been integrated inside the sensing unit, there is no need to use additional modulation circuits and operational amplifiers to process the signal. The signal channels from the pressure sensing insole (6 per foot, 12 on both feet) are connected to the input port of the 16-channel multiplexer module (HC4067, NXP). Under the control of the microcontroller (Arduino UNO, Ivrea, Italy), the multiplexer traverses all the connection channels in turn, and transmits the collected analogue signal to the analogue input port of the microcontroller and passes the built-in ADC (analogue-to-digital converter), which converts the voltage signal into a digital signal for storage. The sensor signal data is recorded in a file on the SD card for offline analysis and evaluation of the performance of the plantar pressure-sensitive insole. To improve the overall ease of use, we designed an Arduino UNO expansion integrated circuit board to integrate all the above modules into the expansion circuit board, as shown in Figure 8. The entire system can be powered by a DC voltage source of 5 V~12 V for power supply, such as a polymer lithium battery.

#### 2.2.4. Plantar Pressure Center

COP is widely used in the study of plantar pressure-related parameters, especially the division of the gait phase, so it can be used as the most direct evaluation parameter for verifying pressure-sensing insoles. During exercise, due to the movement of the body’s center of gravity, the plantar pressure center shows a periodic trend. It moves from the heel to the toe in a single foot and switches back and forth between the two feet. Therefore, based on our design, we refer to the method introduced in the paper [11] to calculate the COP. The COP is divided into $CoP_{x}$ along the inner and outer directions and $CoP_{y}$ in the front and rear directions. The calculation method is shown in Equations (1) and (2):

(1) $$CoP_{x}=\frac{\sum_{i=1}^{6}X_{i}\cdot P_{i}}{\sum_{n=1}^{6}P_{i}},$$

(2) $$CoP_{y}=\frac{\sum_{i=1}^{6}Y_{i}\cdot P_{i}}{\sum_{n=1}^{6}P_{i}}$$

where $X_{i}$ and $Y_{i}$ represent the position of the sensing unit along with the medial/lateral directions and front/rear direction, respectively, as shown in Figure 7. $P_{i}$ represents the signal value of the *i*th sensing unit. It is worth noticing that the plantar pressure center only exists in the standing stage of the leg. Therefore, we define that the center of pressure during the swing stage is located at (0,0) to distinguish the standing and swing phases.

## 3. Experiments and Results

### 3.1. Sensor Characteristic

Using the calibration analysis system introduced above, we can easily carry out the characteristic analysis experiment of a single sensor. The characteristic analysis experiment is mainly to anchor the center of the sensor unit perpendicular to the static load test of the pressure sensing surface. The static load test is defined as a step of 0.025 mm perpendicular to the sensor’s surface and then staying for 3 s to have enough time for stable measurement. The maximum distance is 1 mm (accounting for 14.3% of the thickness of the sensing unit). After the loading process, the unloading process is completed according to the same stepping distance and dwell time until the indenter leaves the surface of the sensing unit. Results of stiffness (force-strain response), sensitivity (resistance-force response) and hysteresis characteristics of a batch of six sensing elements are analyzed.

**Figure 9:**
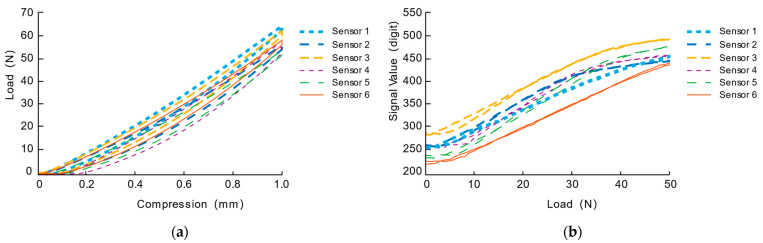
The mechanical and electrical characteristics. (**a**) The mechanical characteristics (**b**) The electrical characteristics.

As shown in Figure 9a, all sensing units exhibit certain mechanical hysteresis characteristics in terms of mechanical characteristics. According to the quantification method of mechanical hysteresis characteristics in the paper [5], that is, through calculation, the ratio of the area enclosed by the loading range and the horizontal axis to the area enclosed by the unloading range and the horizontal axis in the curve is used to quantify the mechanical hysteresis characteristics. The mechanical hysteresis coefficients of each sensor are 0.928, 0.937, 0.947, 0.935, 0.921 and 0.933, respectively. We can observe that the mechanical characteristics of the pressure sensing unit are relatively consistent, which are mainly related to the characteristics of the silicone elastomer inside the sensing unit.

**Figure 10:**
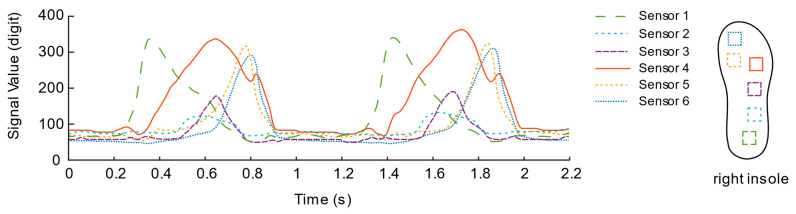
Signal Curve of the first insole solution.

As shown in Figure 9b, there is a certain linear relationship between the sensing signal and the load in terms of electrical characteristics. With the help of MATLAB’s cftool toolbox, we chose to use a polynomial to fit the curve. Here, the relationship curve between signal response and loading force is fitted with a two-order polynomial ($F\left(s\right)=a_{0}+a_{1}s+a_{2}s^{2}$ where *S* represents the loading force, and F represents the output response signal). The fitting results are shown in Table 1. It can be found from Figure 10 that the electrical hysteresis characteristic is almost negligible during loading and unloading. Combining Figure 10 and Table 1, we can observe that the initial sensing signals ($a_{0}$) of the six sensing units under no-load are more or less different. However, from the 0-60N load range result, the sensor’s sensing range is relatively close.

### 3.2. Gait Data Collection

#### 3.2.1. Comparison of Insole Solutions

We evaluate the performance of the two designed induction insole solutions. Preliminary test experiments were carried out on both. The author of this article (male, age 24, height 1.79 m, weight 76.6 kg, shoe size 43) wears two kinds of insoles in indoor corridors according to walking habits, and conducts the following tests: from a natural standing state to a waking state with an average pace, then staying still for a few seconds in the end.

In the experiment, we found that the induction signal has an abnormally negative value. Through observation, we found that the abnormal phenomenon is mainly caused by the asymmetry of the flexible circuit board area in the sensor unit, which causes the internal optical path of the sensor unit to shift when pressure is applied to the circuit around the sensor unit. At this time, the optical path is deflected. The most direct effect of the shift is that the light intensity is reduced compared to the case where the light path is directly facing, which leads to abnormal negative values of the induced signal in the experimental results. Therefore, this is an inevitable feature in the design principle of light-sensitive pressure sensing based on commercial LED light strips in this study. That is, the sensing signal at the center of the sensing unit will be interfered with by the surrounding pressure, but it will be affected by the center of the sensing unit. When the pressure is positive, the pressure signal is in line with theoretical expectations. In response to this phenomenon, we used the linear rectification activation function (Rectified Linear Unit, ReLU) in the neural network to preprocess the signal of the sensing unit and use the negative signal caused by the pressure around the sensing unit under the function of the function. It is filtered, and only the positive part of the sensing signal is retained. The expression equation can be described as:

(3) $$f\left(x\right)=\{\begin{array}{c} \begin{array}{ll} 0 & if\left(x\leq 0\right) \end{array}\\ \begin{array}{cc} x & if(x>0) \end{array} \end{array}\;,$$

**Figure 11:**
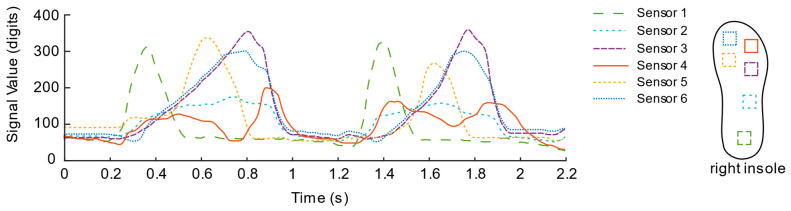
Signal Curve of the second insole solution.

After the rectification activation function is processed, as shown in Figure 10 and Figure 11, the signal curves are drawn from a piece of data intercepted from the walking experiments of the two insole schemes. In contrast, the data curve of solution No. 1 is “messy”, and the mess is mainly reflected in the insufficient regularity of the sensor signal fluctuations located in the toe area.

**Figure 12:**
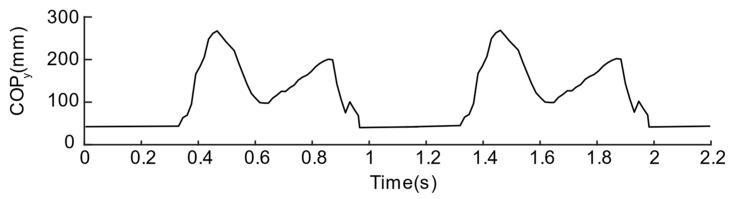
COP values of the first insole solution.

**Figure 13:**
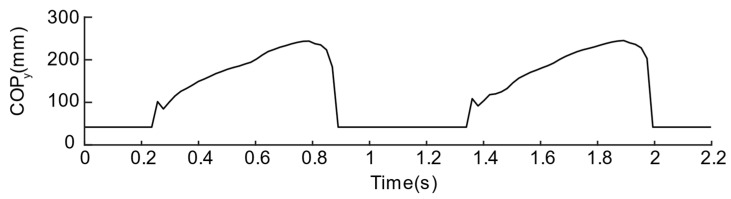
COP values of the second insole solution.

To further compare the quality of the two solutions, the pressure centers are calculated by Equations (1) and (2), as shown in Figure 12 and Figure 13. From the $CoP_{y}$ of the curve of solution No. 1, we can observe that the plantar pressure center during the stance phase is disturbed suddenly and, then, the $CoP_{y}$ of the curve of the solution No. 2 can better reflect that the pressure center is standing. The tendency of the phase is to move from the heel to the toe, therefore the layout of the second solution is regarded as a better sensor layout.

#### 3.2.2. Biped Gait Data Collection

**Figure 14:**
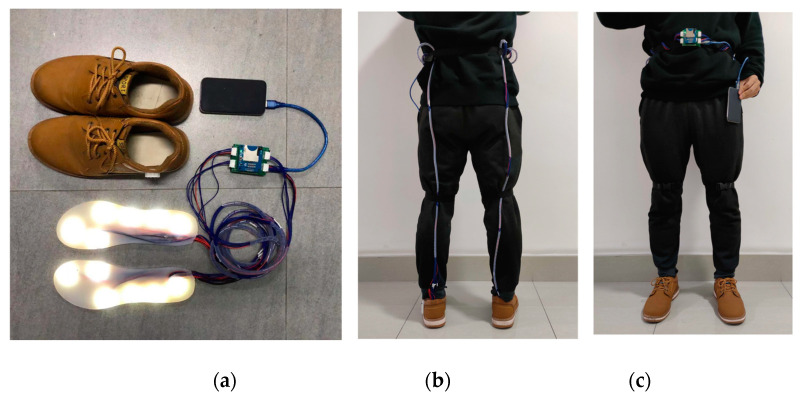
The experimental setup. (**a**) equipment used (**b**) sensing system setup back view (**c**) sensing system setup front view.

We completed the left foot insole production according to the same production process as the right foot insole. Since the circuit system design reserved up to 16 sensing channels for both feet, one only needs to open the left foot sensing channel in the acquisition program to acquire all the sensing channels of the feet (a total of 12 sensing units). The equipment used for the bipedal gait data collection experiment is shown in Figure 14, including a pair of versatile 43 size shoes and a pair of self-designed two-point photoelectric pressure sensor insoles. Before collecting data, the wearer uses a nylon bayonet to fix the wire on the back of the lower limbs, in the manner shown in Figure 14, to avoid the influence of wire swinging on the usual walking movement during walking. The nylon bayonet and cable tie are fixed on the wearer’s waist, and the power bank can be placed in the wearer’s trouser pocket after power is supplied to achieve the minimum hindrance to walking. The gait data of a subject was collected using the device. The subject has never suffered from any disease that hinders walking posture. The experiment process is also from a natural standing state to a waking state with an average pace followed by staying still for a few seconds.

**Figure 15:**
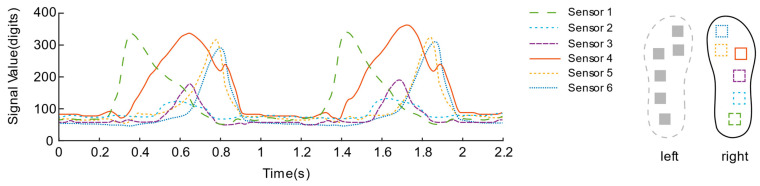
Right foot plantar pressure sensor signal during the bipedal walking experiment.

**Figure 16:**
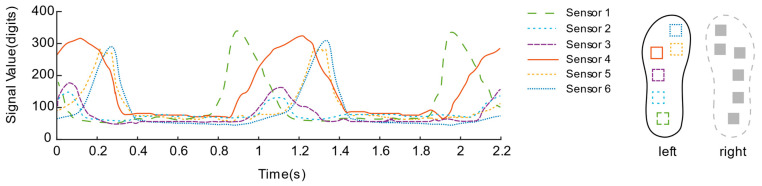
Left foot plantar pressure sensor signal during the bipedal walking experiment.

Figure 15 and Figure 16 describes the dynamic bipedal walking experiment data. Observing the data, each sensor presents a periodic “rest state” and “active state”. During the “active state”, the sensor signals reach their respective peaks in succession. During the “resting state”, all sensors returned to their lower levels, which is consistent with our intuitive impression of the phase of standing support and swing phase during the complete gait cycle of a single leg.

In addition, it can be estimated that the overall walking frequency is about 31 steps per minute from the periodically changing curve. Focusing on the data of each sensor of the insole, we can observe that, during the initial period from the “rest state” to the “active state”, the No. 1 sensor unit located on the heel first senses the pressure and quickly rises to the peak; then, as the pressure of the No. 1 sensor gradually decreases, the pressure of the No. 2 sensors located on the outside of the arch of the foot also change; secondly, the No. 3, No. 4 and No. 5 sensors located on the forefoot and the toes were in the same interval, reaching their respective peaks over a long time; finally, all sensor signals return to a lower level of resting state. Connecting the pressure curves of the left and right feet, we can see that in the short period when the pressure of the right foot is about to enter a lower level, the heel pressure of the left foot has already been generated and rapidly increased to the peak. Similarly, the pressure of the left foot is about to enter a higher level. During the short period of low level, the heel pressure of the right foot has also begun to reach its peak rapidly, which also reveals the “bipedal standing phase” that is not easily noticeable when walking. Both feet are in contact with the ground during walking.

## 4. Discussion

The content shown in this research is mainly focused on the integration of photoelectric sensing technology into the pressure sensing unit to achieve the purpose of sensing plantar pressure information, including the analysis of the photoelectric sensing principle and technology, and the introduction of the technical method realization process. We conducted a test and result analysis of sensor unit characteristics, and performance evaluation during actual use. Here, we will discuss and analyze the results obtained, and make reasonable assumptions and note prospects for further research work.

In this study, in terms of the design of the modular sensing unit, the modular-type sensor provides flexibility for the layout of the pressure sensing insole solution. This modular sensor unit is designed ingeniously and economically. The production of the sensor unit can be completed by using some materials that can be easily purchased from the market. However, as far as the manufacturing method is concerned, the method provided in this article is only for small batches and is a hand-made method, so there are certain defects in the stability and repeatability of the sensor characteristics, which can be found in the sensor characterization section. The result analysis shows that in further research work, if one wants to obtain a more stable and reliable modular sensor unit, it is needed to improve the existing processing technology and manufacturing equipment, and choosing a mature engineering technology may be able to solve this problem. On the other hand, it is worth mentioning that our research introduced a compromised pressure analysis instrument, which can carry out pressure-related calibration test work by building a simple force plate and transforming a desktop-level 3D printer. The design of a programmable calibration instrument derived from the analysis of the mechanical and electrical characteristics of the sensor in this study can also be useful for research teams with limited experimental conditions, that is, who cannot obtain equipment with higher precision and more comprehensive functions.

As for the layout of the pressure sensing insole, in some research work, high-resolution intensive plantar pressure sensing is settled as the research target, such as the research work from [20,23,24]. However, from the perspective of practical application, high resolution means a sharp increase in the number of sensor units. The consequence is that more complex computing processors to deal with the hypermultiplet-channel signal and larger power supply units are required to maintain long-term data recording. However, the plantar pressure distribution is continuous, which means there is information redundancy in the same area. The modular sensor unit can be used to lay out the area with main pressure characteristics (such as the pressure sensing insole layout solutions in this article) to reduce the density of the sensing element. In fact, by adjusting the layout position of the sensing unit, the stability and accuracy of the acquisition of plantar pressure gait data can be improved in a controllable manner as exhibited in this research. On the one hand, the experimental results show that, due to the structural configuration of the sensing unit itself, there exists a phenomenon of “negative pressure” around the sensing unit. We analyzed the cause of this phenomenon and used the rectification activation function to preprocess the negative signal. On the other hand, under normal circumstances, the pressure sensor can better monitor the changes in plantar pressure in the position where the force is more extensive. The sensor unit located in the toe area may not be selected as the pressure sensor unit due to the need to control the stability–sensation area. We mainly focused on the analysis of the results of COP in the front and rear directions, which also showed a specific rule; that is, during the period when the observed leg is in the standing support phase, the movement trend of the center of pressure is to shift from the heel position to the toe position gradually. From this information, the following stages of gait can be preliminarily observed:

(1) Swing stage: the sole hardly exerts a force on the insole, and the total pressure is in a stable state and lower than the standing state.
(2) Heel contact stage: the heel touches the ground and bears weight, and the pressure on the heel area increases significantly.
(3) Intermediate stance phase: the heel no longer bears the same pressure as the heel contact phase, and part of the pressure is transferred to the front foot.
(4) Toe off stage: the body’s center of gravity is almost moved to the other side of the body, the heel is off the ground, and the pressure is mainly concentrated on the forefoot.

However, this article only shows a hand-made/manual operation of modifying the sensor layout. In view of the increasing application of machine learning and other technologies in the engineering field, if the sensor layout can be used as an optimization goal, optimization methods based on machine learning can be employed to perform a much better sensing unit layout for a wider range of plantar pressure distribution information. We believe that the optimization strategy using artificial intelligence algorithms will bring reference significance for better design layout.

What is more, when discussing this research work from the perspective of signal transmission, the current design work adopts the method of transmitting the signal through the cable to obtain the pressure signal. It needs to be admitted that although, in our design, the cables are carefully routed according to the path that does not hinder the movement as much as possible, it is inevitable that there are annoying obstacles such as position displacement and winding during the actual locomotion. Wires will negatively affect the reliability and complexity of wearable devices (usually tied to the human body). Therefore, in further research work, wireless communication protocols (such as WIFI, Bluetooth) are urgently desired for signal transmission. The development of a wireless transmission version of the plantar pressure measurement system can get rid of the annoying winding that hinders movement and improve the integration of the entire sensor measurement system, providing a more convenient interface for further integration into wearable applications.

## 5. Conclusions

The design and application of a simple and reliable plantar pressure data acquisition device is very important for wearable human body assist equipment such as exoskeletons and power prosthesis. In this research, we conducted a novel plantar pressure sensing insole based on photoelectric sensing technology. The innovation of this modular sensing unit focuses on sensing principles, structural design and elastic materials. We introduced how to use low-cost manufacturing methods and materials to fabricate this modular sensor so that other researchers can easily replicate and further develop related research work. The designed modular pressure sensor realizes calibration analysis on a self-designed programmable calibration instrument. The electromechanical performance evaluation experiment proves that the modular pressure sensor has special applicability in pressure sensing. Subsequently, based on the analysis of plantar pressure, we proposed and compared different pressure-sensitive insole layout solutions, and integrated the modular pressure sensor into the pressure-sensitive insole. The dynamic locomotion results showed that the pressure-sensing insole based on the photoelectric effect can capture the deformation of the insole caused by plantar pressure during walking, sense the distribution of plantar pressure and provide reliable gait-related parameters with no interference to the wearer. This research provides a reliable gait information acquisition device for wearable applications such as powered exoskeletons, prosthesis and orthotics.

## Figures and Tables

**Table 1:** Fitting Result of Sensing Unit Electrical Characteristic.

Sensor	Fitted Coefficients			Fitting Effect		0–60 N Load RanΔS
	$a_{0}$	$a_{1}$	$a_{2}$	RMSE	$R^{2}$	
1#	261.2	9.867	−0.105	1.074	0.997	161.3
2#	234.5	5.421	−0.011	2.395	0.992	176.4
3#	281.5	6.517	−0.051	2.753	0.998	169.7
4#	264.7	8.714	−0.091	2.711	0.998	173.4
5#	240.1	8.098	−0.058	2.475	0.996	185.9
6#	225.5	4.974	−0.027	1.713	0.993	178.3

## Data Availability

The datasets generated and/or analysed during the current study are available from the corresponding author on reasonable request.
